# Sleep-Dependent Oscillatory Synchronization: A Role in Fear Memory Consolidation

**DOI:** 10.3389/fncir.2017.00049

**Published:** 2017-07-06

**Authors:** Michael S. Totty, Logan A. Chesney, Phillip A. Geist, Subimal Datta

**Affiliations:** ^1^Laboratory of Sleep and Cognitive Neuroscience, Department of Anesthesiology, Graduate School of Medicine, University of TennesseeKnoxville, TN, United States; ^2^Department of Psychology, College of Arts and Sciences, University of TennesseeKnoxville, TN, United States

**Keywords:** plasticity, coherence, REM sleep, non-REM sleep, theta wave, delta wave, memory processing, neuronal network

## Abstract

Sleep plays an important role in memory consolidation through the facilitation of neuronal plasticity; however, how sleep accomplishes this remains to be completely understood. It has previously been demonstrated that neural oscillations are an intrinsic mechanism by which the brain precisely controls neural ensembles. Inter-regional synchronization of these oscillations is also known to facilitate long-range communication and long-term potentiation (LTP). In the present study, we investigated how the characteristic rhythms found in local field potentials (LFPs) during non-REM and REM sleep play a role in emotional memory consolidation. Chronically implanted bipolar electrodes in the lateral amygdala (LA), dorsal and ventral hippocampus (DH, VH), and the infra-limbic (IL), and pre-limbic (PL) prefrontal cortex were used to record LFPs across sleep-wake activity following each day of a Pavlovian cued fear conditioning paradigm. This resulted in three principle findings: (1) theta rhythms during REM sleep are highly synchronized between regions; (2) the extent of inter-regional synchronization during REM and non-REM sleep is altered by FC and EX; (3) the mean phase difference of synchronization between the LA and VH during REM sleep predicts changes in freezing after cued fear extinction. These results both oppose a currently proposed model of sleep-dependent memory consolidation and provide a novel finding which suggests that the role of REM sleep theta rhythms in memory consolidation may rely more on the relative phase-shift between neural oscillations, rather than the extent of phase synchronization.

## Introduction

It is well-established that sleep plays an important role in learning and memory (Stickgold, 2005; Datta, 2006; Maquet, 2007; Diekelmann and Born, 2010; Ravassard et al., 2016; Li et al., 2017). More specifically, phasic activity during REM sleep and spindle activity during non-REM sleep has been heavily implicated in successful memory consolidation (Datta, 2000; Datta et al., 2004; Girardeau et al., 2009; Fogel and Smith, 2011). These effects on memory have been largely attributed to changes in neuronal plasticity, such as long-term potentiation (LTP), long-term depression (LTD), and other cellular modifications (Bramham et al., 1994; Datta et al., 2008; Shaffery et al., 2012; Dumoulin Bridi et al., 2015; Frank, 2015; Hennies et al., 2016; Ravassard et al., 2016; Li et al., 2017). Despite this knowledge, the mechanisms by which sleep facilitates memory consolidation remains to be completely understood.

Both non-REM and REM sleep are characterized by dominant neural oscillations; however, the purpose of these rhythms also remain elusive. Behavioral state-dependent neural oscillations have been widely implicated in cognitive functions, including learning and memory (Basar et al., 2000; Buzsáki and Draguhn, 2004; Hutchison and Rathore, 2015). Specifically, theta rhythms (4–8 Hz) found throughout the medial prefrontal cortex, amygdala, and hippocampus have been shown to modulate aversive memories and facilitate plasticity (Hyman et al., 2003; Seidenbecher et al., 2003; Likhtik and Gordon, 2014; Harris and Gordon, 2015; Boyce et al., 2016). Similarly, delta rhythms (1–4 Hz) have been implicated in cognitive functions and the coordination of neural network activity, although, evidence for this is scarce (Diekelmann and Born, 2010; Carracedo et al., 2013; Nácher et al., 2013). Coupled with the knowledge of sleep's role in memory, it is logical to assume that these characteristic rhythms may play an important role in memory consolidation.

One particularly interesting phenomenon which may facilitate delta and theta rhythms potential role in learning and memory is inter-regional synchronization. Synchronization of neural oscillations is an intrinsic mechanism by which rhythms facilitate neural communication and plasticity (Fries, 2005; Cannon et al., 2014). It accomplishes this by ensuring network organization by coordinating neural firing, thus modulating the likelihood for successful information transfer and development of LTP or LTD (Fell and Axmacher, 2011; Cannon et al., 2014). In fact, synchronization of theta oscillations has been linked to the retrieval of fearful memories and the successful extinction of fear-related memories (Seidenbecher et al., 2003; Lesting et al., 2013). Furthermore, individual changes in spectral coherence between the medial prefrontal cortex, amygdala, and hippocampus during REM sleep is correlated to the retrieval of fear memories (Popa et al., 2010). Indeed, it appears oscillatory synchronization is an excellent candidate for facilitating memory consolidation; however, more evidence is needed to elucidate this function.

Mechanisms by which sleep favors memory consolidation remains to be poorly understood, particularly at the network level. In this study, we used a fear learning paradigm to investigate delta and theta network dynamics of the cortico-limbic system across vigilance states. Here we show that cortico-limbic REM sleep theta rhythms are not only highly synchronized between regions, but that it is the most synchronous slow rhythm of any vigilance state within this circuit. Additionally, during non-REM sleep, delta rhythms are highly synchronized between the pre-limbic prefrontal cortex (PL) and the lateral amygdala (LA), dorsal and ventral hippocampus (DH; VH). Furthermore, many changes in oscillatory synchronization observed during non-REM and REM following fear conditioning and extinction were counter-intuitive considering previous literature which suggests bidirectional roles for the PL and infra-limbic prefrontal cortex (IL). Finally, we show that the mean phase difference of synchronization between the LA and VH during REM sleep predicts changes in freezing after cued fear extinction. This suggests that the role of oscillatory synchronization in memory consolidation may rely more on the relative phase-shift between neural oscillations, rather than the extent of phase synchronization.

## Materials and methods

### Subjects and housing

Experiments were performed on eight naive adult male Sprague-Dawley rats (Charles River, Wilmington, MA). Upon arrival, the rats were housed individually with free access to food and water and maintained on a 12-h light/dark cycle. All experiments took place in the daytime during the light phase. Animals were handled once daily for a minimum for 5 min to reduce potential stress induced by experimental handling. Habituation handling began 1 week prior to surgery and continued until recording sessions. Procedures were performed in accordance with the National Institutes of Health Guide for the Care and Use of Laboratory Animals and were approved by University of Tennessee Animal Care and Use Committee (Protocol Number: #2349-UTK). On the basis of ensuring the validity of experimental results, efforts were made to reduce the number of animals used in our experiments and to minimize any possible suffering by the animals. All experiments are conducted in compliance with the ARRIVE guidelines (Kilkenny et al., 2010).

### Electrode implantation

**Figure 1:**
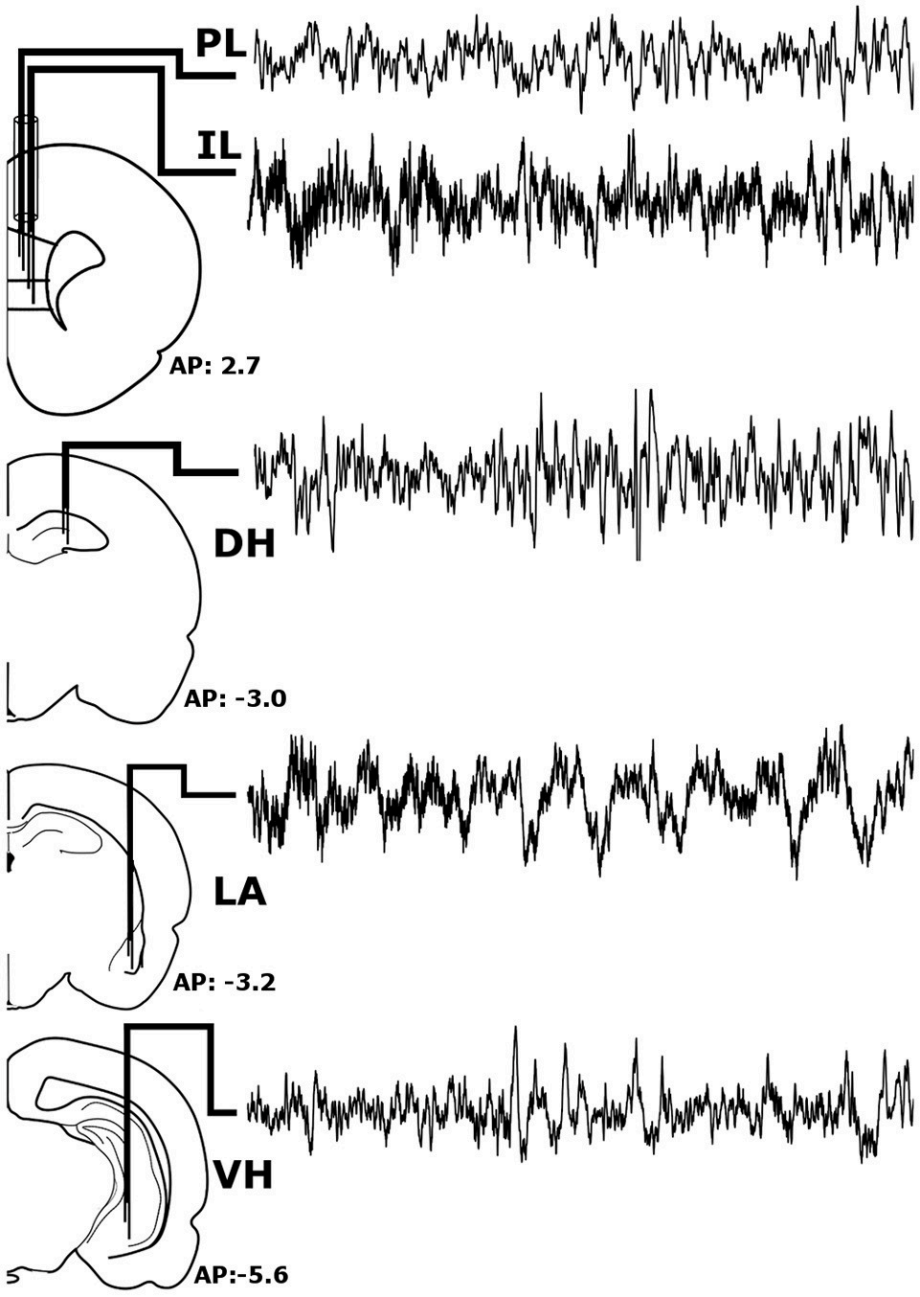
Stereotaxic Implantation. Bipolar electrodes were chronically implanted in the pre-limbic and infra-limbic prefrontal cortex (PL; IL), dorsal and ventral hippocampus (DH; VH), and the lateral amygdala (LA) to record local field potentials. The raw signals pictured above were recorded simultaneously from a single animal during wakefulness and represent roughly 6 s of recording time.

Prior to experimentation, rats were chronically implanted with custom-made, tungsten bipolar electrodes (0.2 mm tip separation). All surgical procedures were performed stereotaxically under aseptic conditions. Animals were anesthetized with a mixture of ketamine (70 mg/kg, i.p.) and xylazine (6.25 mg/kg i.p.), placed into a stereotaxic apparatus, and secured using blunt rodent ear bars. Once the scalp was incised, three burr holes were drilled for screw placement: one anchor screw and two ground electrode screws. Then, the bipolar electrodes were chronically implanted within the LA (−3.2 mm AP, +5.0 mm ML, −9.0 mm DV), VH (−5.6 mm AP, +4.6 mm ML, −8.0 mm DV), and DH (−3.0 mm AP, +2.2 mm ML, −3.8 mm DV), as shown in Figure 1. To record from the both the IL and PL, two bipolar electrodes were threaded through a single stainless-steel guide tube with a separation of 0.3 mm and chronically implanted (+2.7 mm AP, +0.5 mm ML, −4.8/−5.3 DV for PL/IL). All electrodes were secured to the skull with dental acrylic, crimped to mini-connector pins, and placed in a plastic headstage. Immediately following surgery, animals were administered saline (5 cc, s.c.) to prevent dehydration and buprenorphine (0.05 mg/kh, s.c.; Ben Venue Laboratories, Bedford, OH, USA) to alleviate potential postoperative pain.

### Behavioral protocol

**Figure 2:**
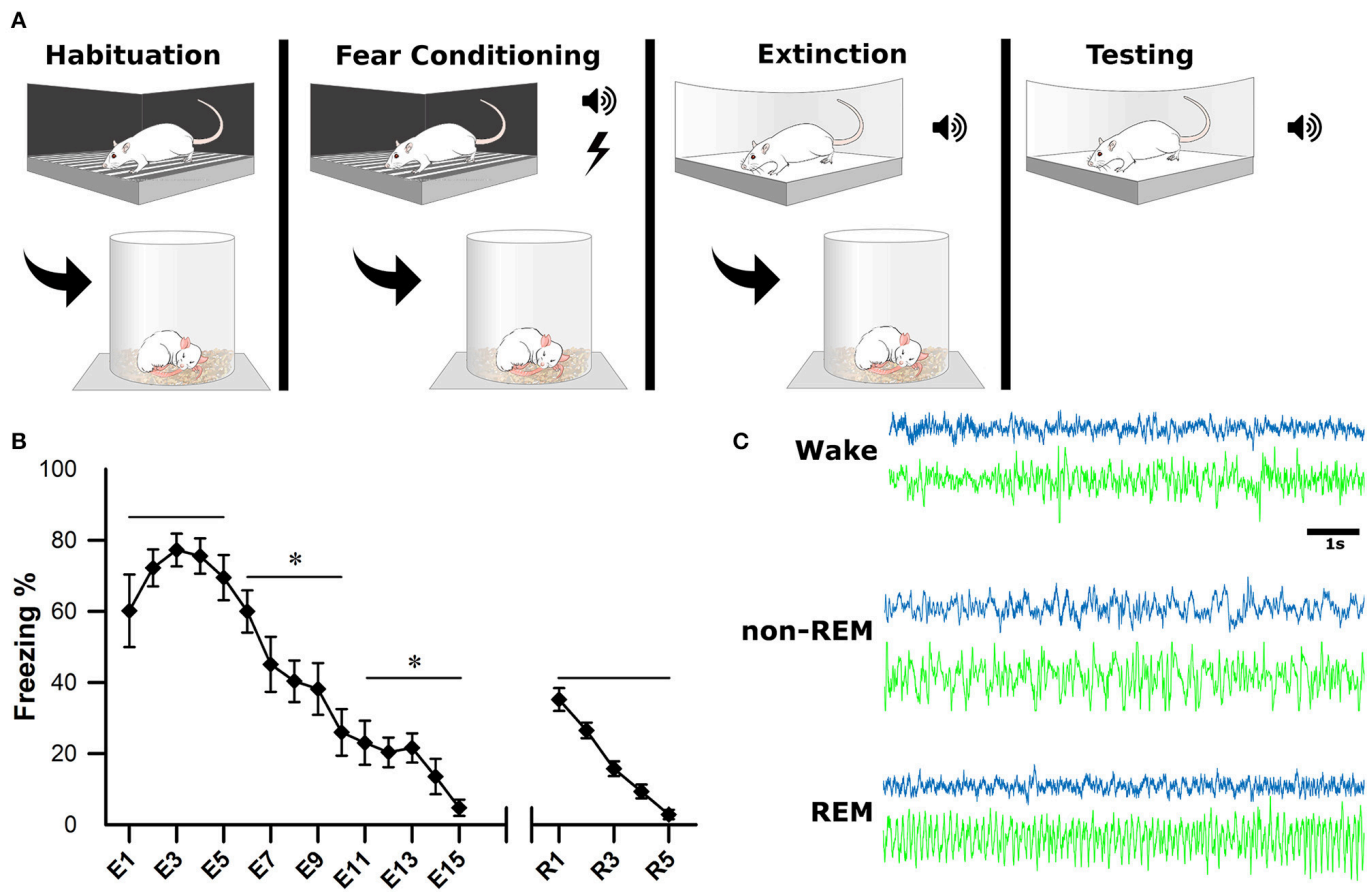
Experimental Design. **(A)** One the first day, animals were habituated to the fear conditioning chamber for 15 min. On the second day, they were subjected to fear conditioning which consisted of three, 10-s tones followed by a 1-s footshock. On the third day, they were placed in a novel context for fear extinction. This consisted of replaying the conditioned tone 15 times (E1–E15), with 2-min inter-stimulus intervals. The final day, animals were tested for successful extinction of fear, which consisted of five more replays of the conditioned tone (R1–R5). Sleep-wake activity was recorded via EEG for 4 h following each of the first 3 experimental days. **(B)** Behavioral measurements of fear were calculated as a percentage of time spent freezing during tone replays. For statistical analysis, trials were grouped into pairs of five. ^*^denotes that the below group of five trials was statistically different from the previous (left to right) *p* < 0.001. As a group, animals successfully acquired and extinguished cued fear memory. **(C)** Electrophysiological sleep-wake activity was later scored based on cortical (blue) and hippocampal (green) electrodes.

After a postsurgical recovery period of at least 7 days, animals were habituated to the recording apparatus and sleep chambers. On the first day of experimentation (baseline, BL), animals were habituated to a fear-conditioning chamber (ShuttleFlex Shuttle Box, Accuscan Instruments, Inc., Columbia, Ohio, USA) with a metal grid floor for 15 min (Figure 2A). On day 2, the animals were returned to the chamber for cued fear conditioning (FC). This consisted of three, 10-s tones (conditioned stimulus, CS; 4 kHz, 80 dB), each co-terminating with a 1 s foot-shock (0.5 mA) with 2-min inter-stimulus intervals (ISI). Day 3, animals were extinguished of cued fear by placing them in a novel context and replaying the CS 15 times with the same 2 min ISI; this time without foot-shock (extinction, EX). The final day tested the recall of the extinction memory. Animals were placed back into the novel context from the day before and five more CS were played with 2-min ISI (Testing).

Immediately following each of the first 3 days of behavioral testing (BL, FC, EX), rats were placed in sleep chambers, connected to the recording apparatus, and allowed 4 h of undisturbed sleep-wake activity. Behavioral measurements of fear (i.e., freezing) were recorded as a video file for offline analysis of extinction and extinction recall (days 3 and 4). Freezing was calculated as percentage during each CS presentation (Figure 2B).

### Electrophysiological recordings

Electrophysiological recordings took place in sleep chambers following days 1–3 of behavioral testing through the use of a multichannel amplifier system (Grass Model 15 Neurodata Amplifier System, Astro-Med Inc.; West Warwick, RI, USA). A swivel recording apparatus was used to maintain the head plug connection while allowing rats to move freely. Local field potentials (LFPs) from each electrode pair were high-pass filtered at 0.5 Hz to mitigate any movement-related artifacts and acquired at 1,000 Hz. After testing each day, data were moved to a computer for offline sleep scoring and electrophysiological analyses.

### Sleep scoring

Sleep scoring was performed manually by experts using SleepSign software (Kissei Comtec Co., Ltd, Matsumoto, Japan) with an epoch length of 10 s, as described previously (Datta, 2002, 2007). Vigilance states were defined by the following criteria (Figure 2C): wakefulness (Wake): low voltage (50–80 μV) and fast (30–50 Hz) cortical oscillations and the absence of prominent, lasting theta (4.5–10 Hz) oscillations in the hippocampus; non-rapid eye movement sleep (non-REM): high-voltage (200–400 μV) slow waves (1–15 Hz) in the cortical EEG; rapid-eye movement sleep (REM): low voltage (50–100 μV) and fast (20–40 Hz) cortical oscillations and theta oscillations in the hippocampus.

### Data analysis

After all data were down sampled to 512 Hz to reduce computation time, 10 epochs of each wakefulness, non-REM, and REM sleep were chosen in a pseudorandom fashion from each animal. Phase-locking value (PLV) and spectrograms were computed using complex Morlet wavelets, as detailed elsewhere (Cohen, 2014). Frequencies were extracted from epochs at each integer from 1 to 30 Hz. The total number of cycles per wavelet was chosen to be 20, due to increased frequency precision and the lack of any temporal events being examined. PLV was chosen over other methods as it utilizes only the phase characteristics, and thus, is not influenced by amplitude. Additionally, unlike spectral coherence, PLV does not rely on stationarity and enables accurate estimations of oscillatory synchronization (Lowet et al., 2016). PLV was computed as previously described (Lachaux et al., 1999; Cohen, 2014) where θ*(t,n)* is the instantaneous phase difference Φx(*t,n*) − Φy(*t,n*) at each time point and *T* is the total number of time points.

(1) $$PLV=\;\frac{1}{T}\left|\sum_{t=1}^{T}e^{i\theta (t,n)}\right|$$

In essence, if phase differences do not deviate over time the trial is highly synchronous with a mean resultant length (MRL) near 1. If the phase differences are completely random over time, the trial is asynchronous with a mean result length near 0. This creates one value for each subsequent trial.

To determine the preferred phase differences within subjects and across trials, phase differences at each time point were averaged using the *circ_mean* function of the Circular Statistics Toolbox (Berens, 2009). The preferred phase angles within subjects were then correlated to the change in percent freezing from the end of EX to the following extinction testing day by use of the *circ_corrcl* function. All of the above analyses were conducted using custom written MATLAB scripts (MathWorks, Inc., Massachusetts, USA).

### Histology

After experimentation was completed, animals were deeply-anesthetized with a mixture of ketamine (100 mg/kg) and xylazine (10 mg/kg) and then were perfused transcardially with 1x PBS (pH 7.4) followed by 10% formalin. Brains were then dissected and placed in a 10% formalin solution for 24 h followed by a 30% sucrose solution for a minimum of 48 h. After the post-fix period, brains were sectioned on a cryostat (−20°C) at 40-μm thickness, mounted on microscope slides, and stained with cresyl violet (Acros Organics, Fisher Scientific, Waltham, Massachusetts) to confirm electrode placement.

### Statistical analysis

Brown-Forsythe and Bartlett's test were conducted to confirm data homogeneity. A one-way analysis of variance (ANOVA) was used to compare both power and PLV changes in each vigilance state across experimental days (IBM SPSS Version 22.0, IBM Corp. Armonk, NY). Additionally, the same test was used to compare PLV across vigilance states. Tukey's *post-hoc* tests were used to determine individual differences between experiment days, with a significance threshold of *p* < 0.05.

## Results

Unfortunately one animal was lost during experimentation and another was lacking a high quality signal for the DH; therefore, the following reports the results for seven animals (10 epoches each; *n* = 70) except for DH results (*n* = 60). Additionally, elevated IL-PL synchrony can be seen in Figures **4**–**7** due to unavoidable volume conduction. Despite this, statistically significant changes are still found across experimental days. All statistics not listed in the text below can be found in Tables 1, 2.

**Table 1:** Significant differences in delta and theta synchrony across vigilance states.

**ID**	**EEG pair**	**Frequency band**	**Type of test**	***F*; *P*-value**	**DoF**	**Mean ± SE**
a	IL-PL	Delta	Two-way ANOVA	**11.9; 0.000**	2, 207	
			R vs. NR	^**^		R: 0.46 ± 0.019
			R vs. W	ns		NR: 0.57 ± 0.028
			NM vs. W	^***^		W: 0.42 ± 0.018
		Theta	Two-way ANOVA	**4.94; 0.008**	2, 207	
			R vs. NR	ns		R: 0.46 ± 0.027
			R vs. W	ns		NR: 0.49 ± 0.027
			NM vs. W	^**^		W: 0.39 ± 0.019
b	IL-DH	Theta	Two-way ANOVA	**7.04; 0.001**	2, 177	
			R vs. NR	^**^		R: 0.31 ± 0.014
			R vs. W	^*^		NR: 0.26 ± 0.008
			NM vs. W	ns		W: 0.27 ± 0.007
c	IL-LA	Delta	Two-way ANOVA	**4.25; 0.016**	2, 207	
			R vs. NR	^*^		R: 0.29 ± 0.009
			R vs. W	ns		NR: 0.34± 0.015
			NM vs. W	ns		W: 0.31 ± 0.010
		Theta	Two-way ANOVA	**4.30; 0.015**	2, 207
			R vs. NR	ns		R: 0.28 ± 0.007
			R vs. W	^*^		NR: 0.26 ± 0.006
			NM vs. W	ns		W: 0.25 ± 0.008
d	IL-VH	Theta	Two-way ANOVA	**6.58; 0.002**	2, 207	
			R vs. NR	ns		R: 0.28 ± 0.008
			R vs. W	^**^		NR: 0.26 ± 0.006
			NM vs. W	ns		W: 0.25 ± 0.006
e	PL-DH	Delta	Two-way ANOVA	**7.47; 0.001**	2, 177	
			R vs. NR	^**^		R: 0.28 ± 0.006
			R vs. W	ns		NR: 0.33 ± 0.012
			NM vs. W	^**^		W: 0.29 ± 0.008
		Theta	Two-way ANOVA	**8.57; 0.000**	2, 177	
			R vs. NR	^***^		R: 0.32 ± 0.011
			R vs. W	^**^		NR: 0.26 ± 0.009
			NM vs. W	ns		W: 0.27 ± 0.009
f	PL-LA	Delta	Two-way ANOVA	**15.39; 0.000**	2, 207	
			R vs. NR	^***^		R: 0.29 ± 0.008
			R vs. W	ns		NR: 0.37 ± 0.012
			NM vs. W	^***^		W: 0.30 ± 0.009
		Theta	Two-way ANOVA	**4.04; 0.019**	2, 207	
			R vs. NR	ns		R: 0.28 ± 0.007
			R vs. W	^*^		NR: 0.26 ± 0.008
			NM vs. W	ns		W: 0.25 ± 0.007
g	PL-VH	Delta	Two-way ANOVA	**5.45; 0.005**	2, 207	
			R vs. NR	^**^		R: 0.28 ± 0.007
			R vs. W	ns		NR: 0.32 ± 0.009
			NM vs. W	ns		W: 0.29 ± 0.009
		Theta	Two-way ANOVA	**21.72; 0.000**	2, 207	
			R vs. NR	^***^		R: 0.29 ± 0.007
			R vs. W	^***^		NR: 0.24 ± 0.006
			NM vs. W	ns		W: 0.24 ± 0.006
h	DH-LA	Theta	Two-way ANOVA	**12.82; 0.000**	2, 177	
			R vs. NR	^***^		R: 0.34 ± 0.012
			R vs. W	^***^		NR: 0.27 ± 0.009
			NM vs. W	ns		W: 0.28 ± 0.012
i	DH-VH	Theta	Two-way ANOVA	**48.529; 000**	2, 177	
			R vs. NR	^***^		R: 0.39 ± 0.015
			R vs. W	^***^		NR: 0.26 ± 0.008
			NM vs. W	ns		W: 0.27 ± 0.009
j	LA-VH	Theta	Two-way ANOVA	**5.09; 0.007**	2, 207	
			R vs. NR	^**^		R: 0.44 ± 0.017
			R vs. W	^*^		NR: 0.37 ± 0.016
			NM vs. W	ns		W: 0.38 ± 0.018

*Displayed in this table are the significant findings for delta and theta PLV across vigilance states. F- and P-values are for the two-way ANOVA and subsequent Tukey's post-hoc comparisons. Bold numbers highlight F- and P-values. Data were acquired during the 4-h sleep-wake recordings following experimental testing*.

* *p < 0.05*;

** *p < 0.01*;

*** *p < 0.001*.

**Table 2:** Significant changes in delta and theta synchrony in non-REM and REM sleep.

**ID**	**EEG pair**	**Frequency band**	**Type of test**	***F*; *P*-value**	**DoF**	**Mean ± SE**
**NON-REM SLEEP**						
a	IL-DH	Delta	Two-way ANOVA	**6.81; 0.001**	2, 177	
			BL vs. FC	^*^		BL: 0.28 ± 0.010
			BL vs. EX	ns		FC: 0.33 ± 0.015
			FC vs. EX	^**^		EX: 0.27 ± 0.009
b	PL-LA	Delta	Two-way ANOVA	**4.0; 0.019**	2, 207	
			BL vs. FC	ns		BL: 0.37 ± 0.012
			BL vs. EX	ns		FC: 0.39 ± 0.014
			FC vs. EX	^*^		EX: 0.34 ± 0.013
		Theta	Two-way ANOVA	**3.6; 0.029**	2, 207	
			BL vs. FC	^*^		BL: 0.26 ± 0.008
			BL vs. EX	ns		FC: 0.29 ± 0.009
			FC vs. EX	ns		EX: 0.27 ± 0.008
c	PL-VH	Delta	Two-way ANOVA	**3.8; 0.024**	2, 207	
			BL vs. FC	ns		BL: 0.32 ± 0.009
			BL vs. EX	ns		FC: 0.34 ± 0.013
			FC vs. EX	^*^		EX: 0.30 ± 0.011
d	DH-LA	Theta	Two-way ANOVA	**5.0; 0.008**	2, 177	
			BL vs. FC	^*^		BL: 0.27 ± 0.009
			BL vs. EX	ns		FC: 0.24 ± 0.007
			FC vs. EX	^*^		EX: 0.27 ± 0.009
e	LA-VH	Delta	Two-way ANOVA	**3.4; 0.034**	2, 207	
			BL vs. FC	ns		BL: 0.37 ± 0.015
			BL vs. EX	ns		FC: 0.41 ± 0.019
			FC vs. EX	^*^		EX: 0.35 ± 0.016
**REM SLEEP**						
f	PL-DH	Theta	Two-way ANOVA	**4.9; 0.009**	2, 177	
			BL vs. FC	**ns**		BL: 0.32 ±0.011
			BL vs. EX	ns		FC: 0.29 ± 0.011
			FC vs. EX	^**^		EX: 0.34 ± 0.012
g	PL-LA	Delta	Two-way ANOVA	**4.1; 0.018**	2, 207	
			BL vs. FC	ns		BL: 0.29 ± 0.008
			BL vs. EX	ns		FC: 0.31 ± 0.010
			FC vs. EX	^*^		EX: 0.27 ± 0.006
h	PL-VH	Theta	Two-way ANOVA	**4.5; 0.012**	2, 207	
			BL vs. FC	ns		BL: 0.29 ± 0.007
			BL vs. EX	^*^		FC: 0.30 ± 0.007
			FC vs. EX	^*^		EX: 0.32 ± 0.009
i	DH-VH	Delta	Two-way ANOVA	**5.1; 0.007**	2, 177	
			BL vs. FC	ns		BL: 0.29 ± 0.007
			BL vs. EX	ns		FC: 0.31 ± 0.011
			FC vs. EX	^**^		EX: 0.27 ± 0.008

*Displayed in this table are the significant findings for changes in delta and theta PLV across during non-REM and REM sleep. Data were acquired during the 4-h sleep-wake recordings following experimental testing. F- and P-values are for the two-way ANOVA and subsequent Tukey's post-hoc comparisons. Bold numbers highlight F- and P-values*.

* *p < 0.05*;

** *p < 0.01*.

### Comparison of synchrony during vigilance states

**Figure 3:**
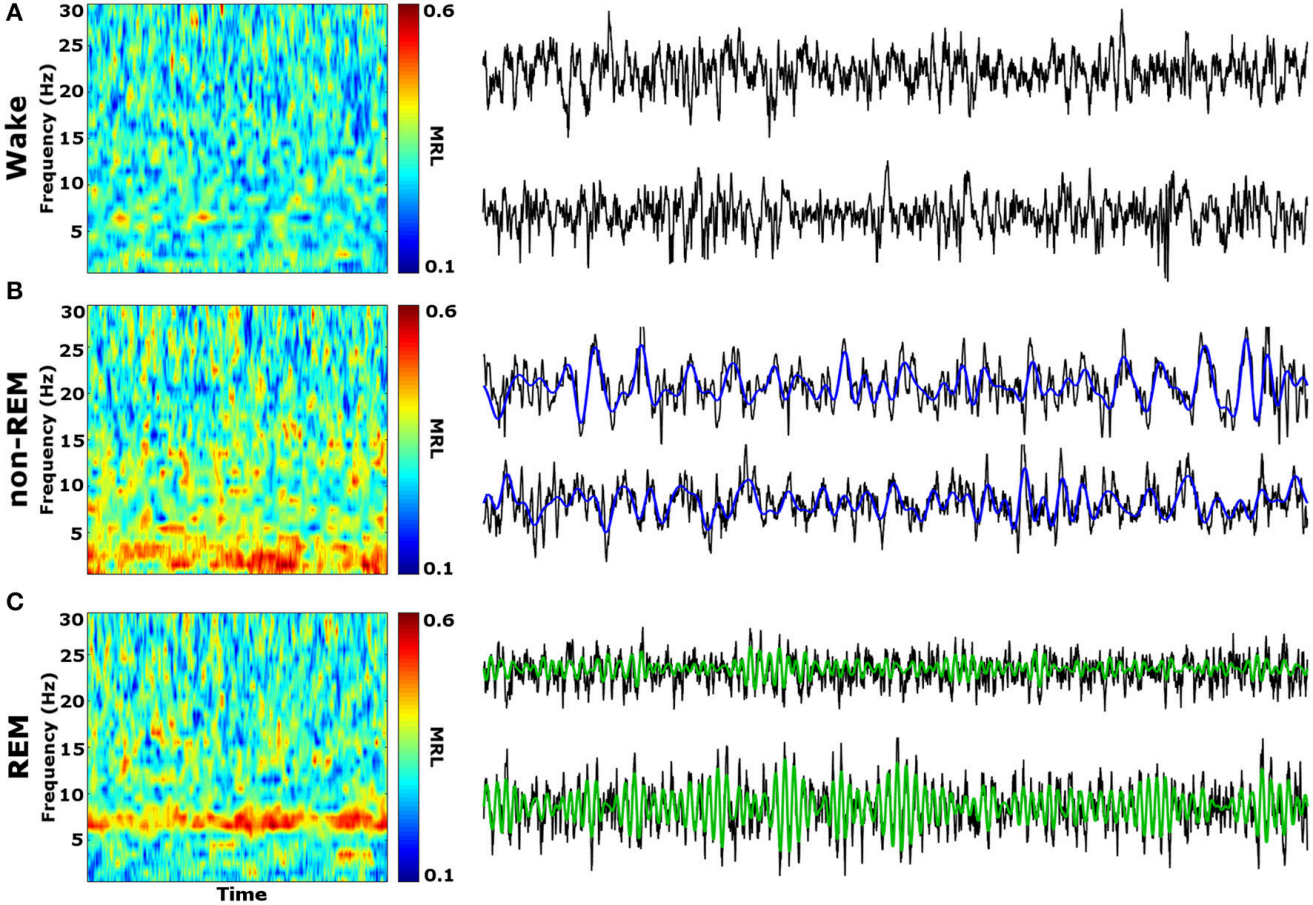
Qualitative Spectrograms of Oscillatory Synchrony across Vigilance States. Example spectrograms displaying typical synchrony (MRL) during vigilance states and their respective LFPs. Although phase-locking was seldom observed during wakefulness **(A)**, delta oscillation (blue filtered LFP) phase-locking was found to be prominent during non-REM sleep **(B)**, and theta oscillation (green filtered LFP) phase-locking was prominent during REM sleep **(C)**. LFPs represent 10 averaged epochs from one animal. MRL, mean resultant length; LFPs, local field potentials.

**Figure 4:**
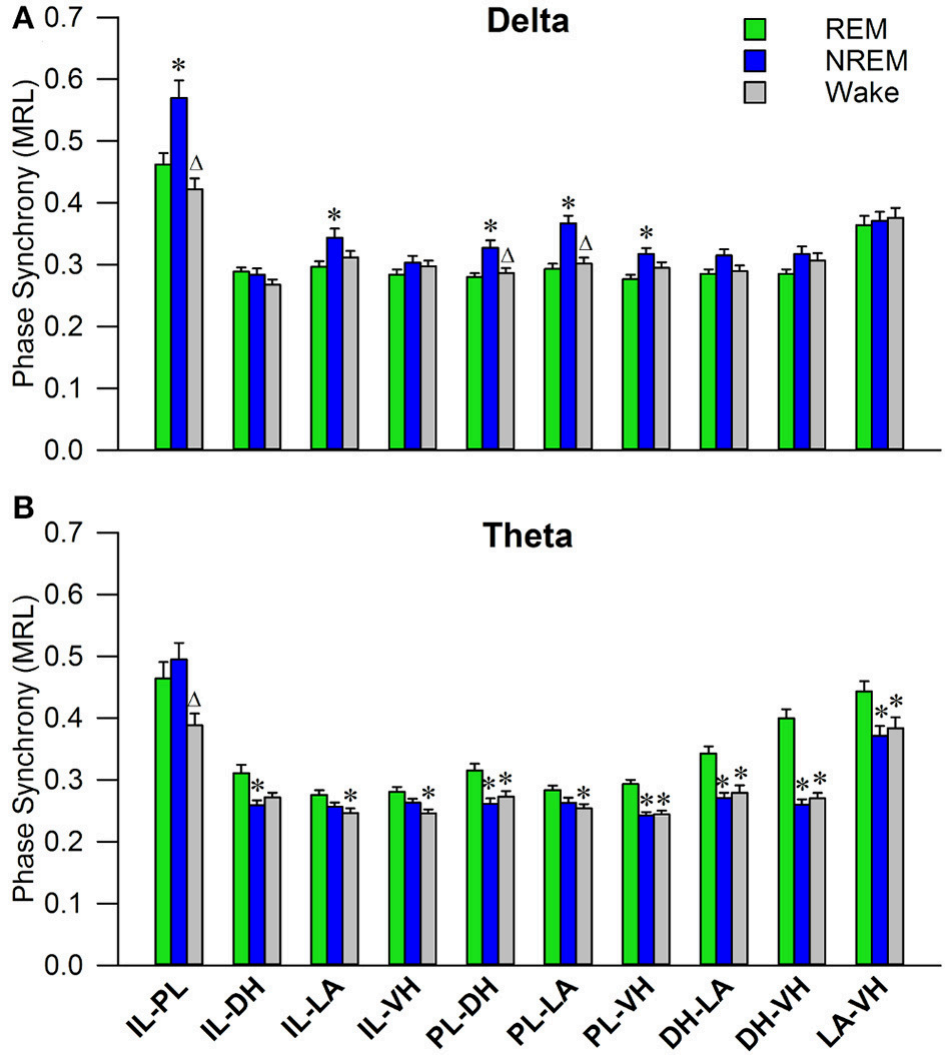
Delta and Theta Synchrony across Vigilance States. Bar plots displaying delta **(A)** and theta **(B)** synchrony across vigilance states, where 1.0 is perfect synchrony and 0 is no synchrony. Delta rhythms appear to synchronize the PL to the DH, LA, and VH during non-REM sleep, while also synchronizing the IL with the PL and LA. Theta rhythms display increased synchrony across every regional pairing except within the cortex (IL-PL). ^*^denotes significant difference (*p* < 0.05) compared to REM, and Δ denotes significant difference (*p* < 0.05) compared to non-REM. PL, pre-limbic; IL, infra-limbic; DH, dorsal hippocampus; LA, lateral amygdala; VH, ventral hippocampus.

Initial qualitative analysis of PLV via spectrogram plotting revealed that wakefulness seldom displayed strong synchrony, whereas non-REM and REM sleep showed synchrony predominantly in the delta and theta frequency bands, respectively (Figure 3). As shown in Figure 4A, for delta rhythms, a one-way ANOVA revealed significant main effects in 5 out of 10 electrode pairs (Table 1): the IL-PL^a^, IL-LA^c^, PL-DH^e^, PL-LA^f^, and PL-VH^g^. Tukey's multiple comparisons test revealed significantly higher PLV during non-REM sleep compared to REM sleep in IL-PL^a^, IL-LA^c^, PL-DH^e^, PL-LA^f^, and PL-VH^g^ electrodes. Higher PLV was also found in non-REM sleep compared to wakefulness in IL-PL^a^, PL-DH^e^, and PL-LA^f^ electrodes. These results show that delta rhythms are more synchronized within the cortex (IL-PL) and between specific cortical and subcortical regions (IL-LA, PL-DH, PL-LA, and PL-VH) during non-REM sleep, compared to both REM sleep and wakefulness.

For theta rhythms, a one-way ANOVA revealed significant main effects in all 10 electrode pairings (Table 1): IL-PL^a^, IL-DH^b^, IL-LA^c^, IL-VH^d^, PL-DH^e^, PL-LA^f^, PL-VH^g^, DH-LA^h^, DH-VH^i^, and LA-VH^j^. As shown in Figure 4B, Tukey's multiple comparisons test revealed significantly higher PLV during REM sleep compared to non-REM sleep in IL-DH^b^, PL-DH^e^, PL-VH^g^, DH-LA^h^, DH-VH^i^, and LA-VH^j^ electrodes. REM sleep PLV was also found to be higher when compared to wakefulness in IL-LA^c^, IL-VH^d^, PL-DH^e^, PL-LA^f^, PL-VH^g^, DH-LA^h^, DH-VH^i^, and LA-VH^j^. Additionally, theta rhythms were found to have higher PLV in non-REM sleep compared to wakefulness in IL-PL^a^ electrodes. In contrast to delta, these results show much stronger synchronization of theta rhythms between subcortical structures during REM sleep. However, theta synchronization is also prominent between cortical and subcortical structures.

### PLV changes in response to fear conditioning and extinction

**Figure 5:**
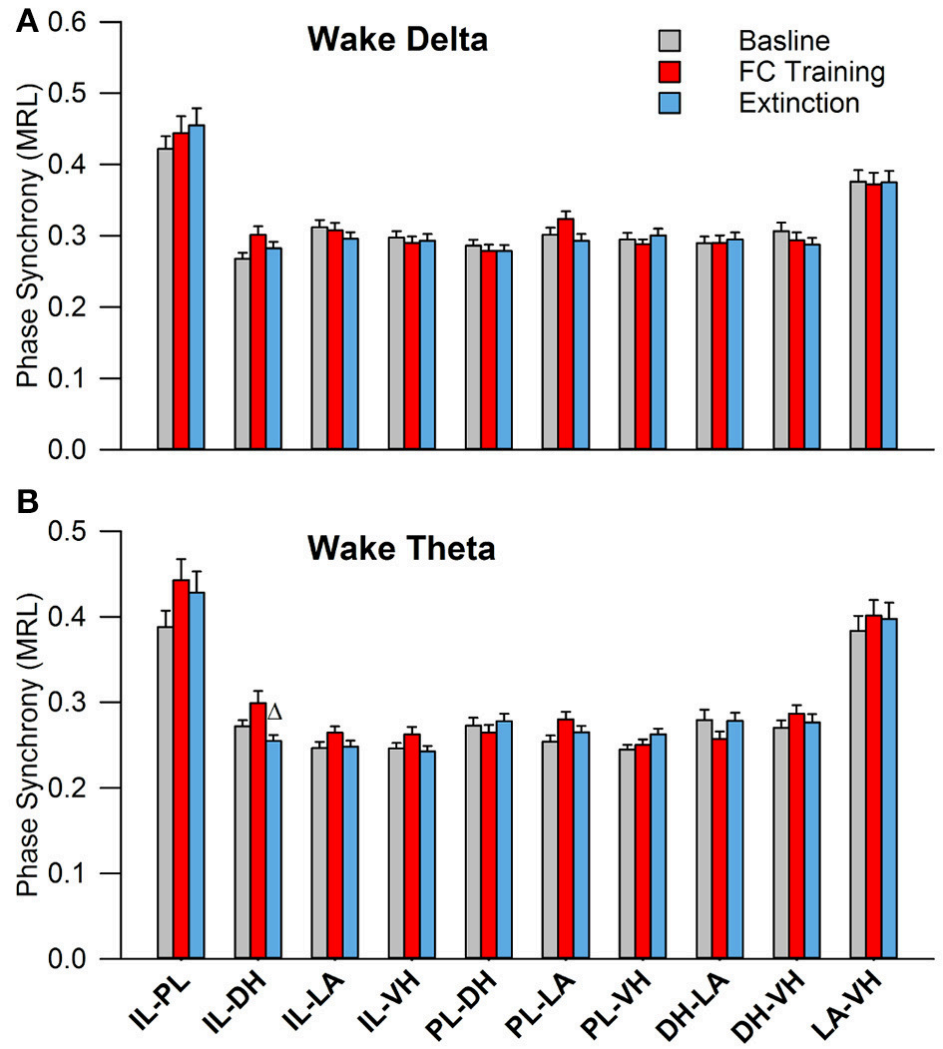
Synchrony during Wakefulness following Fear Conditioning and Extinction. Bar plots displaying delta **(A)** and theta **(B)** synchrony during wakefulness following fear conditioning (red) and extinction (blue), where 1.0 is perfect synchrony and 0 is no synchrony. As expected, neither delta nor theta rhythms change during wakefulness following experimentation. Interestingly, the IL shows significantly increased theta synchrony with the DH following extinction compared to fear conditioning, which contrasts with the current model of the division of labor within the medial prefrontal cortex (Giustino and Maren, 2015). Δ denotes significant difference (*p* < 0.05) compared to fear conditioning. IL, infra-limbic; DH, dorsal hippocampus.

During wakefulness, no significant effects were found for PLV of delta rhythms, as shown in Figure 5A. However, a one-way analysis of variance of PLV of theta rhythms revealed a significant main effect of the IL-DH electrode pairing [Figure 5B; *F*_(2, 177)_ = 4.9, *p* = 0.009]. Tukey's post-test further revealed that PLV decreased following EX compared to FC (FC: 0.30 ± 0.01; EX: 0.25 ± 0.01, *p* < 0.05). Significant effects were not found for any other electrode pairs. These results show limited effects of fear conditioning and extinction on synchrony during wakefulness.

**Figure 6:**
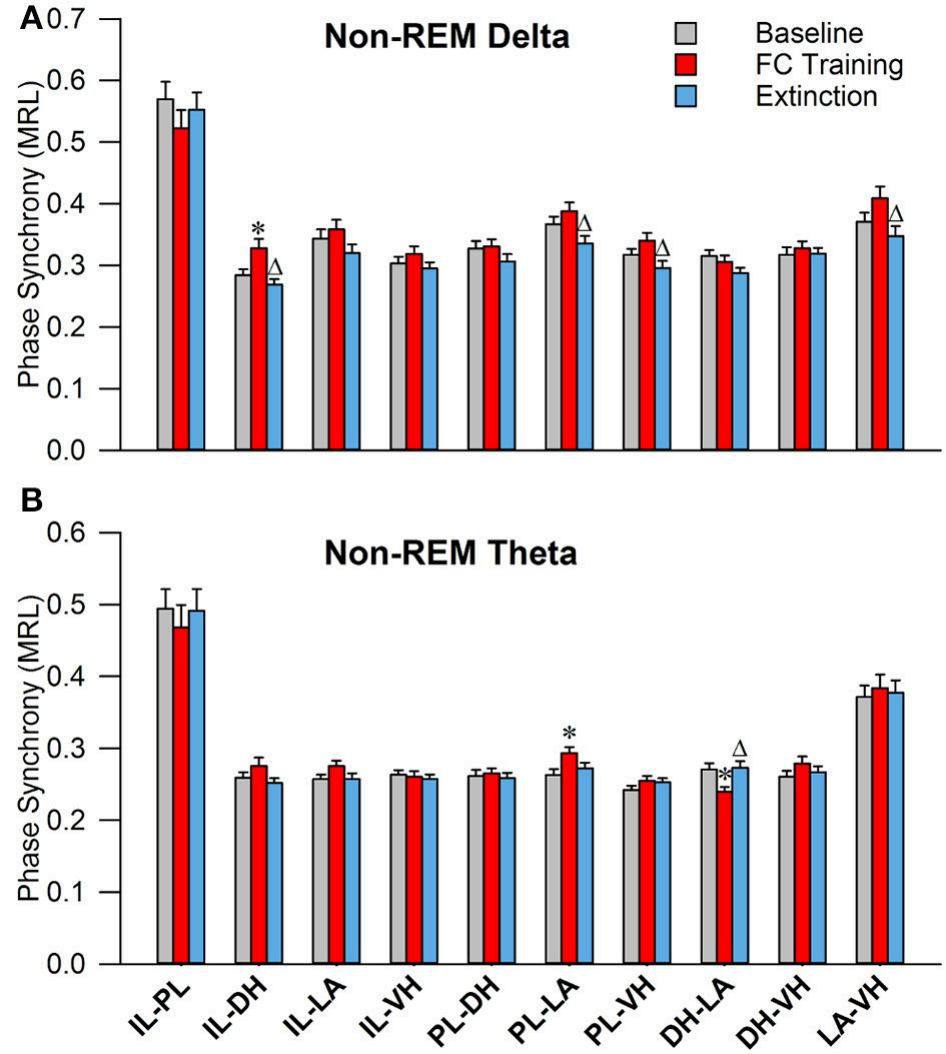
Synchrony during Non-REM Sleep following Fear Conditioning and Extinction. Bar plots displaying PLV of delta **(A)** and theta **(B)** synchrony during non-REM sleep following fear conditioning (red) and extinction (blue). In delta rhythms, IL-DH synchrony increases following fear conditioning, compared to both baseline and extinction. Also, synchrony is increased between the PL-LA, PL-VH, and LA-VH pairings following fear conditioning, compared to extinction. In theta rhythms, we see synchrony increases following fear conditioning between the PL-LA pairings, compared to baseline. Interestingly, we see DH-LA synchrony decrease following fear conditioning, compared to both baseline and extinction. ^*^denotes significant difference (*p* < 0.05) compared to baseline, and Δ denotes significant difference (*p* < 0.05) compared to fear conditioning. IL, infra-limbic; PL, pre-limbic; DH, dorsal hippocampus; LA, lateral amygdala; VH, ventral hippocampus.

As shown in Figure 6A, during non-REM sleep, a one-way ANOVA of PLV of delta rhythms revealed significant main effects of the following (Table 2): IL-DH^a^, PL-LA^b^, PL-VH^c^, and LA-VH^e^ electrode pairings. Compared to BL, Tukey's post-test revealed a significant increase following FC in IL-DH^a^ electrodes. Additionally, compared to FC, a decrease was found following EX in the IL-DH^a^, PL-LA^b^, PL-VH^c^, and LA-VH^e^ electrode pairs. For theta rhythms, a one-way analysis of variance revealed significant main effects of PLV on the PL-LA^b^ and DH-LA^d^ electrode pairs. Tukey's multiple comparisons test revealed that PLV following FC was increased compared to BL in the PL-LA^b^ electrode pair and decreased compared to both BL and EX in DH-LA^d^ pairings. These results show that non-REM sleep revealed greater effects for delta rather than theta synchrony.

**Figure 7:**
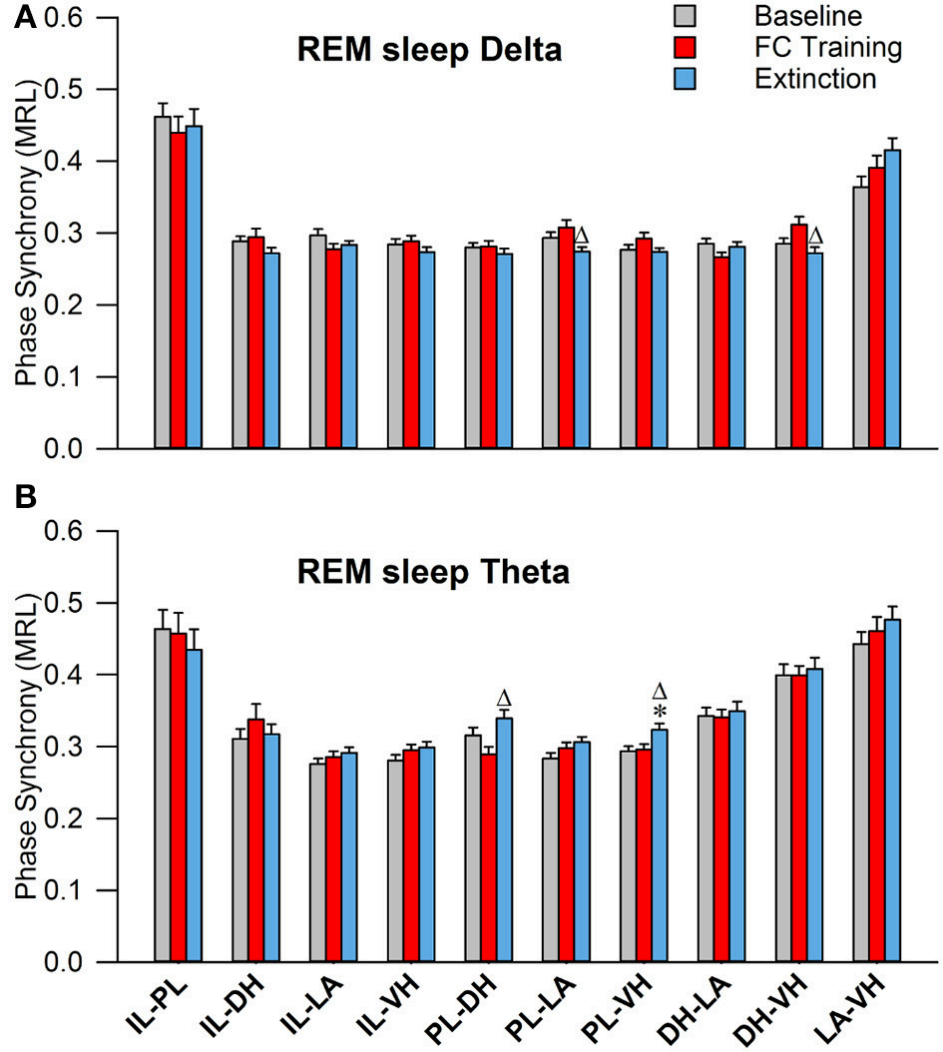
Synchrony during REM Sleep following Fear Conditioning and Extinction. Bar plots displaying PLV of delta **(A)** and theta **(B)** synchrony during REM sleep following fear conditioning (red) and extinction (blue). In delta rhythms, PL-LA and DH-VH synchrony increased following fear conditioning, compared to extinction. In theta rhythms, PL-DH synchrony was increased following extinction, compared to fear conditioning. Additionally, PL-VH synchrony increased following extinction compared to both baseline and fear conditioning. ^*^denotes significant difference (*p* < 0.05) compared to baseline, and Δ denotes significant difference (*p* < 0.05) compared to fear conditioning. PL, pre-limbic; DH, dorsal hippocampus; VH, ventral hippocampus.

During REM sleep, a one-way analysis of variance revealed significant main effects of PLV of delta rhythms in PL-LA^g^ and DH-VH^i^ electrode pairings (Table 2). As shown in Figure 7A, Tukey's post-test revealed that PLV decreased following EX compared to FC in both PL-LA^g^ and DH-VH^i^ pairings. For theta rhythms, a one-way ANOVA revealed significant main effects of PLV on PL-DH^f^ and PL-VH^h^ electrode pairings. Further investigation revealed that PLV increased following EX compared to FC in PL-DH^f^ pairings and that PLV was significantly higher following EX compared to both BL and FC in PL-VH^h^ pairings. These results reveal effects only between a few neural regions for both delta and theta rhythms, which are counter-intuitive considering the highly synchronous activity of theta rhythms during REM sleep compared to the other states of vigilance.

### Mean phase difference correlates to changes in freezing following extinction

**Figure 8:**
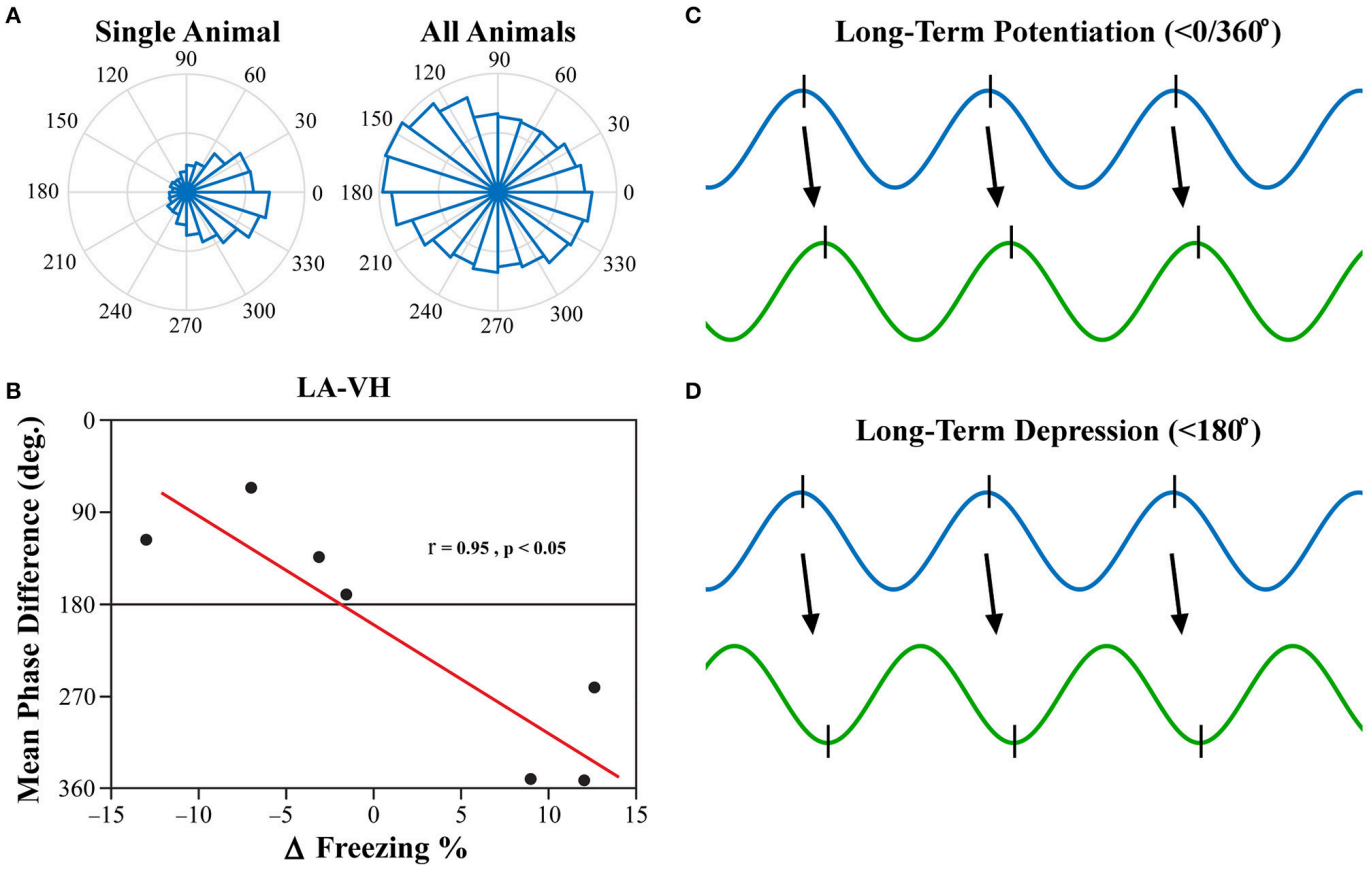
LA-VH Mean Phase Differences of REM Sleep Theta Correlate with Changes in Freezing. Phase angle differences during synchrony were investigated to determine if there were preferred phase differences between regions. REM sleep LA-VH theta was found to display preferred phase differences within single animals, but these preferences varied across animals, as shown by the polar plots **(A)**. Remarkably, these between-subject variations correlated to changes in freezing from the last group of five EX trials to the subsequent group of five Testing trials **(B)**. Animals that showed a decrease in freezing had mean phase differences of just below 180°, whereas those that displayed increased freezing had mean phase differences just below 360/0°. It has previously been demonstrated that neuronal input in the peak of theta oscillations induces LTP **(C)** while input in the trough of theta oscillations induces LTD **(D)** (Huerta and Lisman, 1995; Poe et al., 2000; Hyman et al., 2003). Therefore, we hypothesize that in- and out-of-phase synchronization of theta oscillations may be a mechanism by which synaptic plasticity is modulated during REM sleep, leading to the changes in freezing displayed above. LA, lateral amygdala; VH, ventral hippocampus.

Investigation into the preferred phase differences during vigilance states following FC and EX led to an interesting discovery. Although, strong inter-trial phase clustering was displayed within single subjects, the preferred phase differences across subjects appeared to be more evenly distributed, as displayed in Figure 8A. Using circular statistics, it was found that the mean phase differences of theta rhythms in the LA and VH were significantly correlated to the changes in freezing from EX to Testing during REM sleep (Figure 8B; Pearson's coefficient *R* = 0.954; two-sided *t*-test, *p* = 0.041). Furthermore, visual inspection of Figure 8B reveals decreases in freezing were associated with a near 180° phase difference, whereas increases in freezing were associated with a near 0/360° phase lag. No other significant correlations were found between mean phase differences and changes in freezing between EX and Testing days. The changes in freezing were calculated as the changes from the final group of five trials from EX to the group of five trials on the Testing day.

## Discussion

The present study highlights memory consolidation-associated network interactions during sleep-wake stages. The principal findings of this study are as follows: (1) theta rhythms during REM sleep are highly synchronized between regions; (2) the extent of inter-regional synchronization during REM and non-REM sleep is altered by FC and EX; (3) the mean phase difference of synchronization between the LA and VH during REM sleep predicts changes in freezing after cued fear extinction. These results are discussed to highlight a novel mechanism through which REM sleep theta rhythms may facilitate neural plasticity.

In this study, we investigated the effects of fear learning and extinction on sleep-dependent oscillatory synchronization between five neural regions: the infra-limbic and pre-limbic medial prefrontal cortex (IL; PL), ventral and dorsal hippocampus (VH; DH), and the lateral amygdala (LA). We hypothesized that the levels of sleep-dependent oscillatory synchronization (recorded immediately following each day of experimentation) should increase following fear-related learning, as both sleep and oscillatory synchronization have been implicated in memory processes. In the current model of memory function in sleep, non-REM and REM sleep are responsible for system consolidation and local synaptic modification, respectively (Datta, 2010; Diekelmann and Born, 2010). Although the role of memory in non-REM sleep has been well studied, REM sleep's role is lacking substantial evidence to either confirm or deny the prior theory. For example, tonic theta rhythms within the hippocampus are less synchronized during REM sleep compared to other vigilance states (Montgomery et al., 2008); however, there is also evidence to suggest memory replay during REM sleep (Poe et al., 2000; Louie and Wilson, 2001). In the present study, we show that REM sleep theta rhythms are not only highly synchronous, but they are the most synchronous tonic rhythm observed in the cortico-limbic system during either wake or sleep (Figure 4B). In-line with a recent study (Vijayan et al., 2017), this high measure of functional connectivity is suggestive of inter-regional communication during REM sleep which contrasts with the prior hypothesis that REM sleep is a disentangled state (Diekelmann and Born, 2010). Additionally, non-REM sleep delta rhythms were also revealed to be highly synchronous between many regions (Figure 4A). The medial prefrontal cortex (mPFC) displayed high synchrony between the IL and PL and with the amygdala and hippocampus, which is not surprising considering delta rhythms are most prominent in the cortex during non-REM sleep. Prior evidence for a role of delta rhythm's role in memory is lacking; however, these results suggest that they may play an import role in inter-regional communication during sleep. These results provide unique views of the synchronization of the cortico-limbic system during sleep, particularly for theta oscillations during REM sleep.

In addition to high baseline oscillatory synchronization, changes in oscillatory synchrony provide evidence for a role in memory consolidation. Interestingly, the changes noted in this study are not completely in line with existing literature. The dominant model of the mPFC suggests a division of labor between the PL and IL such that they are responsible for fear expression and suppression, respectively (Sierra-mercado et al., 2010; Giustino and Maren, 2015). Many of the inter-regional dynamics observed in this study support this hypothesis. During non-REM sleep, delta PL synchrony decreased with the LA and VH following EX compared to FC (Figure 6). Additionally, PL-LA delta synchrony decreased during REM sleep (Figure 7). However, many inter-regional dynamics oppose this hypothesis. For instance, increased synchrony between the IL and DH during non-REM sleep following fear conditioning does not support the role of fear suppression for the IL (Figure 6). Additionally, PL theta synchrony with the DH and VH increased following EX during REM sleep (Figure 7). Moreover, many significant effects were found during sleep involving the PL, despite studies implicating the IL, not the PL, in memory consolidation and learning (Laurent and Westbrook, 2009; Sierra-Mercado et al., 2011). It has recently been suggested that the functions of these regions may not be as simple as previously described (Giustino and Maren, 2015); indeed, these results suggest a more complex division of labor. A few changes in synchronization were also noted within subcortical regions, such as REM sleep DH-VH delta (Figure 7A) and non-REM DH-LA delta and DH-LA theta (Figure 6). Although, changes were found, the effects of fear learning and extinction of sleep-dependent oscillatory synchronization were not as profound as we expected. Perhaps these oscillations play a more complex role in memory processes than we previously thought.

Past studies have focused on the precise timing of neuronal firing in relation to specific neural oscillations, otherwise known as temporal coding (Ainsworth et al., 2012). Although, PLV is an excellent, robust measure of oscillatory synchronization, it is simply a measure of the consistency of synchronization over time and leaves out critical information such as preferred phase difference. It has previously been demonstrated that the phase of neural input (peak vs. trough) determines if synapses will be strengthened (LTP) or weakened (LTD) (Huerta and Lisman, 1995; Hyman et al., 2003). Furthermore, it has been shown that novel memories are replayed during REM sleep at the peak of theta oscillations, whereas older memories are replayed at the trough (Poe et al., 2000). Moreover, in-phase synchronization in particular has been hypothesized to facilitate neuronal plasticity (Fell and Axmacher, 2011). In-phase synchronization has largely been the focus in this field of thought, as out-of-phase synchrony should block communication due to neural inputs arriving at the LFPs lowest point of excitability. This, however, does not mean that out-of-phase synchronization would be unable to illicit neuronal firing, but rather, that it would be less likely (but still possible). Thus, we believe that out-of-phase synchronization could play a role in the facilitation of LTD. We show here for the first time, to the best of our knowledge, that the mean phase difference between the LA and VH within subjects predicts individual changes in freezing following fear extinction (Figure 8B). Furthermore, animals that displayed a reduction in freezing following extinction had a <180° phase difference (out-of-phase), whereas animals that displayed increased freezing had a <0/360° phase difference (in-phase). We argue this suggests that in-phase synchrony promotes LTP, whereas out-of-phase synchrony promotes LTD, of this fear-expressing pathway. Thus, we hypothesize that REM sleep theta rhythms modulate synaptic plasticity by dynamically modulating temporal coding and spike-timing dependent plasticity. This is intuitive because if regional oscillations are ~180° out-of-phase, the pre-synaptic neuron will have the greatest probability of depolarizing the post-synaptic neuron in the trough of its respective local oscillation, inducing LTD (Figure 8D). Conversely, regions with near zero phase lag will result in the post-synaptic neuron firing in the peak of its respective local oscillation, successfully strengthening this pathway (Figure 8C).

Admittedly, there are two glaring questions within this finding: (1) why were the phase differences not more directly related to 180° and 0/360°; (2) and why was this only observed for one electrode pairing? The first question can likely be answered by the conduction delay of the action potential. Unless two regions have a mutual input or lie in close proximity to one another, an exactly 0° phase lag (or exactly 180° lag) should not be expected (Fell and Axmacher, 2011). Conduction velocities can be extraordinarily high, but it still takes time for the input to arrive, causing the resulting local oscillation to lag behind. Additionally, it is not necessary for action potentials to arrive at exactly the maxima and minima of a neural oscillation to invoke changes in neural plasticity (Fell and Axmacher, 2011). For the second question, a recent study by Kitamura et al. demonstrated that recent fear memories are stored primarily in the hippocampus and amygdala, not the mPFC (Kitamura et al., 2017). They showed that recall of recent fear memories used a pathway comprised of the hippocampus, entorhinal cortex, and amygdala, and it was nearly 2 weeks before mPFC engrams became active. This is relevant as our testing of the proposed consolidation of a recent fear-related memory resulted in synchrony between the LA and VH, but neither the IL nor PL. Further, the VH has been implicated more closely with emotional processes similar to the LA, unlike the DH which performs primarily cognitive functions (Fanselow and Dong, 2010); thus, it makes sense from this perspective that the LA would be synchronized with the VH instead of the DH. Despite these findings, we acknowledge that at this stage we are unable to claim this without absolute certainty. Future studies should incorporate single-cell recordings that can more precisely investigate temporal coding and spike-timing dependent plasticity.

In conclusion, theta oscillations during REM sleep are highly synchronized inter-regionally throughout the cortico-limbic system, suggesting that theta oscillations are a prime candidate for long-range system consolidation. Though the changes in both delta and theta synchrony were smaller than expected, the displayed changes in inter-regional synchronization in response to emotional learning further supports this hypothesis. Moreover, we hypothesize that LTP and LTD may be modulated by the phase-shift of synchronized REM sleep theta rhythms. This data provides a novel perspective on the role of neuronal rhythms found during non-REM and REM sleep in emotional memory consolidation. This study highlights the importance of a better understanding of neuronal network dynamics in order to completely elucidate sleep's role in behavioral plasticity. Further, we hope that these findings will provide a useful foundation for future investigations.

## Authors contributions

MT: designed the study, performed experiments, analyzed data, and wrote manuscript. LC and PG: performed experiment. SD: designed the study and co-wrote manuscript.

### Conflict of interest statement

The authors declare that the research was conducted in the absence of any commercial or financial relationships that could be construed as a potential conflict of interest.
